# Autophagy attenuates the catabolic effect during inflammatory conditions in nucleus pulposus cells, as sustained by NF-κB and JNK inhibition

**DOI:** 10.3892/ijmm.2015.2280

**Published:** 2015-07-10

**Authors:** KANG XU, WEIJIAN CHEN, XIAOFEI WANG, YAN PENG, ANJING LIANG, DONGSHENG HUANG, CHUNHAI LI, WEI YE

**Affiliations:** 1 Experimental Center of the Surgery, Sun Yat-sen Memorial Hospital, Sun Yat-sen University, Guangzhou, Guangdong 510120, P.R. China; 2 Department of Orthopedics, The Second People's Hospital of Guangdong Province, Guangzhou, Guangdong 510080, P.R. China; 3 Department of Spinal Surgery, Sun Yat-sen Memorial Hospital, Sun Yat-sen University, Guangzhou, Guangdong 510120, P.R. China

**Keywords:** autophagy, intervertebral disc degeneration, nuclear factor κB signaling pathway, nucleus pulposus, inflammatory

## Abstract

Proteoglycan degradation contributing to the pathogenesis of intervertebral disc (IVD) degeneration is induced by inflammatory cytokines, such as tumor necrosis factor-α (TNF-α) and interleukin-1β (IL-1β). Cell autophagy exists in degenerative diseases, including osteoarthritis and inter-vertebral disc degeneration. However, the autophagy induced by TNF-α and IL-1β and the corresponding molecular mechanism appear to be cell-type dependent. The effect and mechanism of autophagy regulated by TNF-α and IL-1β in IVDs remains unclear. Additionally, the impact of autophagy on the catabolic effect in inflammatory conditions also remains elusive. In the present study, autophagy activator and inhibitor were used to demonstrate the impact of autophagy on the catabolic effect induced by TNF-α. A critical role of autophagy was identified in rat nucleus pulposus (NP) cells: Inhibition of autophagy suppresses, while activation of autophagy enhances, the catabolic effect of cytokines. Subsequently, the autophagy-related gene expression in rat NP cells following TNF-α and IL-1β treatment was observed using immunofluorescence, quantitative polymerase chain reaction and western blot analysis; however, no association was present. In addition, nuclear factor κB (NF-κB), c-Jun N-terminal kinase (JNK), extracellular signal-regulated kinases and p38 mitogen-activated protein kinase inhibitors and TNF-α were used to determine the molecular mechanism of autophagy during the inflammatory conditions, and only the NF-κB and JNK inhibitor were found to enhance the autophagy of rat NP cells. Finally, IKKβ knockdown was used to further confirm the effect of the NF-κB signal on human NP cells autophagy, and the data showed that IKKβ knockdown upregulated the autophagy of NP cells during inflammatory conditions.

## Introduction

Lower back pain (LBP) is one of the most common musculoskeletal disorders, and ~40% of LBP involves degeneration of the intervertebral discs (IVDs) (1). IVDs are composed of two distinct components: The inner gel-like core nucleus pulposus (NP) and the outer firm annulus fibrosus (AF). Relying upon a delicate balance between matrix synthesis and degradation, the extracellular matrix (ECM), including collagen and proteoglycans, undergoes a process of remodeling in normal IVDs. However, in degenerative IVDs the net increase of matrix-degrading proteinase activity disrupts the normal balance and leads to the breakdown of ECM (2).

Inflammatory cytokines, such as tumor necrosis factor-α (TNF-α) and interleukin-1β (IL-1β), are highly expressed in degenerative IVDs and contribute to a degenerative IVD phenotype by inhibiting the production of ECM (3,4). While not directly degrading the IVD, TNF-α and IL-1β act indirectly by promoting the production of degradative enzymes, such as matrix metalloproteinase (MMP) and a disintegrin and metalloproteinase with thrombospondin motifs (ADAMTS) (5–7).

Autophagy is involved in the control of cell death (8). Macroautophagy (hereafter referred to as autophagy) is a vacuolar lysosomal degradation pathway for organelles and cytoplasmic macromolecules (9). It occurs during tissue and organ formation and has a critical role in the pathogenesis of degenerative diseases, such as osteoarthritis and Alzheimer's disease (10,11). In IVDs, autophagy is also present and associated with the increased pathological process of IVD degeneration in rats. Furthermore, autophagy of AF cells may be secondary to endoplasmic reticulum stress (12,13). In addition, Shen *et al* (14) reported that the autophagy of rat AF cells was induced by serum deprivation *in vitro* and that IL-1β upregulated serum deprivation-induced autophagy in a dose-dependent manner. Ma *et al* (15) revealed that compression activated autophagy in NP cells and that compression-induced autophagy was closely associated with intracellular reactive oxygen species production.

In inflammatory conditions the inhibition of autophagy increased the expression of OA-like genes, such as *MMP13* and *ADAMTS5*, while the induction of autophagy suppressed these genes (16,17). Regardless, the effect of autophagy on the catabolic effect of inflammatory cytokines in NP cells remains unclear. Additionally, TNF-α and IL-1β activated the autophagy of chondrocyte cells and murine fibrosarcoma L929 cells (16,18). Nuclear factor κB (NF-κB) and mitogen-activated protein kinase (MAPK) signaling pathways, including extracellular signal-regulated kinases (ERK), c-Jun N-terminal kinase (JNK) and p38 MAPK signaling pathways, are involved in the autophagy process. However, the molecular mechanism appears to be cell-type dependent. Certain studies have identified those signaling pathways as a potent negative regulator of autophagy, while others have shown them to be a potent positive regulator (18–25). Thus far, the impact and molecular mechanisms of cytokines on the autophagy of NP cells have remained elusive.

The overall objective of the present study was to demonstrate the impact of autophagy in catabolic factors regulation by cytokines and the effect and mechanism of cytokines, TNF-α and IL-1β, on autophagy in NP cells.

## Materials and methods

### Reagents

3-Methyladenine (3-MA; autophagy inhibitor), rapamycin (autophagy activator), SM7368 (NF-κB inhibitor), PD98059 (ERK inhibitor), SP600125 (JNK inhibitor) and SB203580 (p38 MAPK inhibitor) were purchased from Calbiochem (Danvers, MA, USA). TNF-α and IL-1β were obtained from Peprotech, Inc. (Rocky Hill, NJ, USA). Beclin-1 (#3495), LC3 (#12741), GAPDH (#2118) antibody and rabbit immunoglobulin G (IgG) conjugated with horseradish peroxidase were obtained from Cell Signaling Technology, Inc. (Beverly, MA, USA), while the MMP3 (#ab52915) and COX2 (#ab179800) antibodies were purchased from Abcam (Cambridge, UK). NF-κB reporter construct, psPAX2, pMD2.G, pRL-TK and pLKO.1 plasmids were kindly provided by Dr D Xiao (Nanfang Medical University, Guangzhou, China) (26). *IKKβ* shRNA (TRCN0000018917) was purchased from Dharmacon, Inc. (Lafayette, CO, USA), and the knockdown sequence was ATGTTCAAGATATGAACCAGC.

### Isolation, culture and treatment of NP cells

Consistent with the Institutional Review Board guidelines of Sun Yat-sen University (Guangzhou, China), human NP tissue samples of Pfirrmann grades 1–2 (27) were obtained from two female thoracolumbar fracture patients undergoing spinal fusion. Informed consent for sample collection was obtained from each patient. All the Sprague-Dawley rats were obtained from the Laboratory Animal Center of Sun Yat-sen University. Experimental procedures were approved by the Animal Care and Use Committee of Sun Yat-sen University.

NP cells were isolated as described by Ye *et al* (28). For isolation of rat NP cells, following euthanization by an overdose of pentobarbital (100 mg/kg body weight), the lumbar IVDs of Sprague-Dawley rats, aged 2 months, were collected. Subsequently, NP tissues were separated from AF tissues under the microscope. Later, the NP tissues from the same rats were cut into small pieces, digested with 0.2% pronase medium (Sigma, St. Louis, MO, USA) for 1 h and subsequently cultured in Dulbecco's modified Eagle's medium (DMEM; Gibco-BRL, Gaithersburg, MD, USA) with 10% fetal bovine serum (FBS) and antibiotics (100 U/ml penicillin and 100 U/ml streptomycin) at 37°C in a 5% CO_2_ incubator. The medium was refreshed every 3 days. Subsequent to reaching 80% confluence, the NP cells were treated with TNF-α or IL-1β and at corresponding time-points the cell RNA or protein extraction was performed. The inhibitor or activator was added 1 h before TNF-α or IL-1β.

### Immunofluorescence microscopy

Rat NP cells were plated in 96-well plates (6×10^3^ cells/well). After the treatment with TNF-α and IL-1β for 24 h, NP cells were fixed with 4% paraformaldehyde, permeabilized with 1% Triton X-100 for 10 min and blocked with phosphate-buffered saline (PBS) containing 5% FBS serum for 1 h at room temperature. The cells were subsequently incubated with antibodies against LC3-II antibody (1:200; Cell Signaling Technology, Inc.) at 4°C overnight. The following day, NP cells were washed with PBS and were incubated with Alexa Fluor 488-conjugated anti-rabbit (Invitrogen Life Technologies, Carlsbad, CA, USA) secondary antibody at a dilution of 1:100 for 1 h and 50 *µ*M propidium iodide for 15 min at room temperature. The images were captured with a fluorescent microscope.

### Transfections and dual-luciferase reporter assay

Rat NP cells were seeded in 48-well plates (4×10^4^ cells/well) with 2% Opti-MEM. The following day, 250 ng of NF-κB reporter construct and 250 ng pRL-TK plasmids were premixed with the transfection reagent, Lipofectamine 2000 (Invitrogen Life Technologies) and were co-transfected cells. At 48 h after transfection, the cells were treated with TNF-α (50 ng/ml) or IL-1β (10 ng/ml) for 24 h, and subsequently the cells were harvested. Firefly and *Renilla* luciferase activities were measured by a dual-luciferase reporter assay (Promega Corporation, Madison, WI, USA). All the luciferase assays were performed in triplicate and every experiment was repeated ≥3 times.

### IKKβ knockdown

As described previously (28), HEK 293T human embryonic kidney cells at a density of 3×10^6^ cells/10-cm plate were seeded in DMEM with 10% heat-inactivated FBS. Approximately 24 h later, cells were transfected with 9 *µ*g of shRNA control sequence or *IKK* shRNA plasmids, along with 6 *µ*g psPAX2 and 3 *µ*g pMD2.G. The transfection medium was replaced with DMEM with 10% heat-inactivated FBS 16 h later. At 48 and 60 h after transfection, the plasmid medium containing lentiviral particles was harvested from HEK 293T cells. Subsequently, a virus solution replaced the medium in the plate-seeded human NP cells at a density of 0.5×10^6^ cells/10-cm plate, along with 6 mg/ml polybrene. Five days later, cells were harvested for protein extraction.

### Reverse transcription-quantitative polymerase chain reaction (RT-qPCR)

Total RNA was extracted with TRIzol reagent (Invitrogen Life Technologies) following the manufacturer's instructions. Single-stranded cDNA templates were prepared from 2,000 ng total RNA using SuperScript III Reverse Transcriptase (Invitrogen Life Technologies). Template cDNA and gene-specific primers were added to Fast SYBR Green Master mix (Applied Biosystems, Foster City, CA, USA) and mRNA expression was quantified using the 7900HT Fast Real-Time PCR System (Applied Biosystems). *Hprt* was used to normalize the expression. Each sample was analyzed in duplicate. All the primers used were synthesized by Shanghai Sangon Biological Engineering Technology & Services Co., Ltd. (Shanghai, China).

The primers were as follows: *MMP2* sense, GGTGGTGGTCACAGCTATTT and antisense, CCAGCCAGTCCGATTTGAT; *MMP3* sense, CAGGGAAAGTGACCCACATATT and antisense, CGCCAAGTTTCAGAGGAAGA; *MMP9* sense, CCCAACCTTTACCAGCTACTC and antisense, GTCAGAACCGACCCTACAAAG; *ADMATS4* sense, GGAGATCGTGTTTCCAGAGAAG and antisense, CAAAGGCTGGTAATCGGTACA; *COX2* sense, TCAACCAGCAGTTCCAGTATC and antisense, GTGTACTCCTGGTCTTCAATGT; and *Hprt* sense, GCTGACCTGCTGGATTACAT and anti-sense, CCCGTTGACTGGTCATTACA.

### Western blot analysis

Following the treatment, the plates with NP cells were placed on ice immediately. The condition medium was collected with corresponding collection tubes, and cells were washed with ice-cold PBS twice and collected with scrapers. Following centrifugation (10,000 × g), the cells were treated with lysis buffer, including 1X Protease Inhibitor Cocktail (Roche Diagnostics GmbH, Mannheim, Germany), NaCl (5 mM), NaF (200 *µ*M), Na_3_VO_4_ (200 *µ*M) and dithio-threitol (0.1 mM). Subsequently, total cell proteins (30 ng) qualified with bicinchoninic acid reagent were resolved on 10–15% SDS-polyacrylamide gels and transferred by electroblotting to PVDF membranes (Bio-Rad, Hercules, Hercules, CA, USA). The membranes were blocked with 5% non-fat dry milk in 50 mM Tris (pH 7.6), 150 mM NaCl and 0.1% Tween-20, and incubated overnight at 4°C with anti-Beclin-1 (1:1,000), anti-LC3 (1:1,000), anti-COX2 (1:1,000), anti-MMP3 (1:500), anti-IKKβ (1:1,000) and anti-GAPDH (1:3,000). Finally, the membranes were incubated in anti-serum against rabbit or mouse IgG conjugated with horseradish peroxidase (Cell Signaling Technology, Inc.) (1:1,000–5,000) for 1 h and subsequently treated with ECL Plus according to the manufacturer's instructions (Amersham Pharmacia Biotech, Umeå, Sweden). Blot intensity was determined by densitometric analysis using Kodak 1D 3.6 software (Kodak, Rochester, NY, USA). Beclin-1, LC3-II, COX2, MMP3 and IKKβ protein expression data were normalized to GAPDH expression, which was the internal control.

### Acridine orange staining for NP cell autophagy

As a marker of autophagy, the volume of the cellular acidic compartment was visualized by acridine orange staining; acridine orange staining of rat NP cells was performed as described by Paglin *et al* (29) and Wang *et al* (30). Rat NP cells (6×10^3^ cells/well) were seeded in 96-well plates, and at 50% confluence the cells were treated with TNF-α and SM7368 or SP600125. The cells were incubated with acridine orange (1 *µ*g/ml) 24 h later at 37°C. After 30 min, the acridine orange was removed and a confocal microscope was used to immediately detect the autophagy; 488 nm excitation light, and 520–530 nm (green) and 650 nm (red) emission light were used. The acidic autophagic vacuoles exhibit red fluorescence, whereas the cytoplasm and nucleus of the stained cells exhibit bright green fluorescence.

### Statistical analysis

All the experiments were performed in triplicate. All the data are presented as the mean ± standard error. Differences between the groups were analyzed by one-way analysis of variance. P<0.05 were considered to indicate a statistically significant difference.

## Results

### Autophagy decreases the catabolic effect of IL-1β

**Figure 1:**
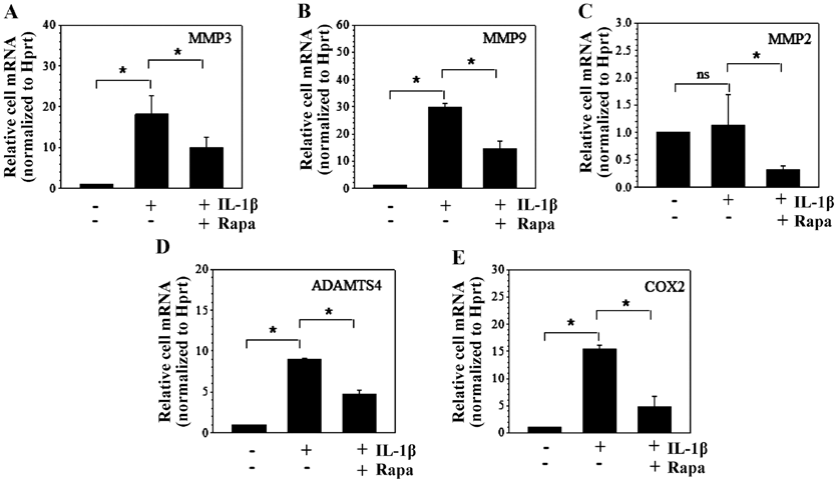
Autophagy is an important regulator of the catabolic effects induced by interleukin-1β (IL-1β) in nucleus pulposus (NP) cells. mRNA expression of (A) matrix metalloproteinase 3 (*MMP3*) and (B) *MMP9* in NP cells was induced by IL-1β and significantly repressed by additional rapamycin (Rapa). (C) *MMP2* mRNA expression was not regulated by IL-1β, however, it was suppressed by additional Rapa. (D) A disintegrin and metalloproteinase with thrombospondin motif 4 (*ADAMTS4*) and (E) *COX2* mRNA expression in NP cells induced by IL-1β repressed by additional Rapa. Data represent mean + standard error of three independent experiments. ^*^P<0.05; ns, not significant.

Rapamycin and 3-MA are an autophagy activator and inhibitor, respectively. To investigate the impact of autophagy on the catabolic effect, rapamycin (1 *µ*M) or 3-MA (5 mM) was added 1 h before 10 ng/ml IL-1β. Compared to the control group, IL-1β stimulation led to ~18- and 30-fold increases of *MMP3* and *MMP9* mRNA expression in NP cells, respectively, and additional rapamycin treatment resulted in decreases of ~10- and 16-fold, respectively (Fig. 1A and B). Although IL-1β had no effect on *MMP2* mRNA expression, rapamycin significantly suppressed *MMP2* mRNA expression when IL-1β was present (Fig. 1C). In addition, *ADAMTS4* and *COX2* mRNA expression were induced to ~9- and 15-fold by IL-1β treatment and reduced to ~4.5- and 5-fold following the additional rapamycin stimulation, respectively (Fig. 1D and E). Since the baseline expression of catabolic factors, such as MMP3 and ADAMTS5, was not too high, we did not observe the effects of rapamycin alone.

### Autophagy decreases the catabolic effect of TNF-α

**Figure 2:**
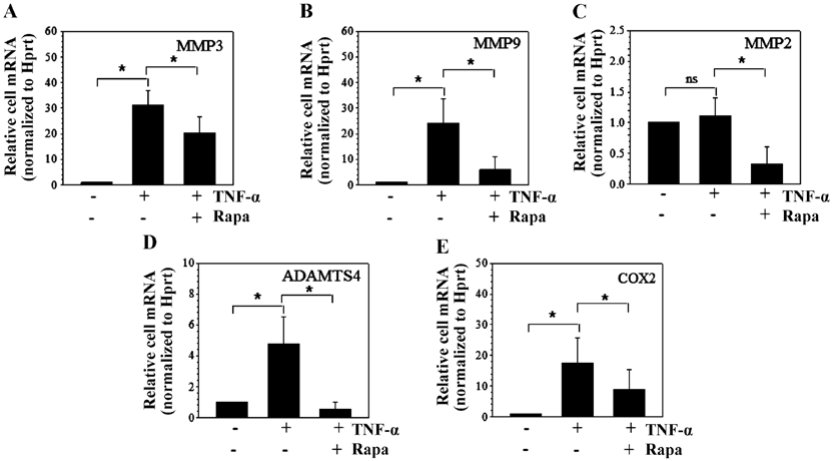
Autophagy represses the catabolic effects induced by tumor necrosis factor-α (TNF-α) in nucleus pulposus (NP) cells at the mRNA level. mRNA expression of (A) matrix metalloproteinase 3 (*MMP3*) and (B) *MMP9* in NP cells was induced by TNF-α and markedly repressed by additional rapamycin (Rapa). (C) *MMP2* mRNA expression was downregulated by Rapa but was not regulated by TNF-α. (D) A disintegrin and metalloproteinase with thrombospondin motifs (*ADAMTS4*) and (E) *COX2* mRNA expression in NP cells upregulated by TNF-α and subsequently suppressed by additional Rapa. Data represent mean + standard error of three independent experiments. ^*^P<0.05; ns, not significant.

Similarly, rapamycin (1 *µ*M) or 3-MA (5 mM) was used to observe the effect of autophagy on the catabolic effect induced by 50 ng/ml TNF-α. TNF-α stimulation led to the increase of *MMP3* and *MMP9* mRNA expression in NP cells (Fig. 2A–C). However, additional rapamycin treatment decreased *MMP3* and *MMP9* mRNA expression (Fig. 2A and B). As with IL-1β treatment, *MMP2* mRNA expression was not regulated by TNF-α, however, rapamycin reduced *MMP2* mRNA expression when TNF-α was present (Fig. 2C). Additionally, *ADAMTS4* and *COX2* mRNA expression was upregulated by TNF-α, and was markedly suppressed by additional rapamycin treatment (Fig. 2D and E).

**Figure 3:**
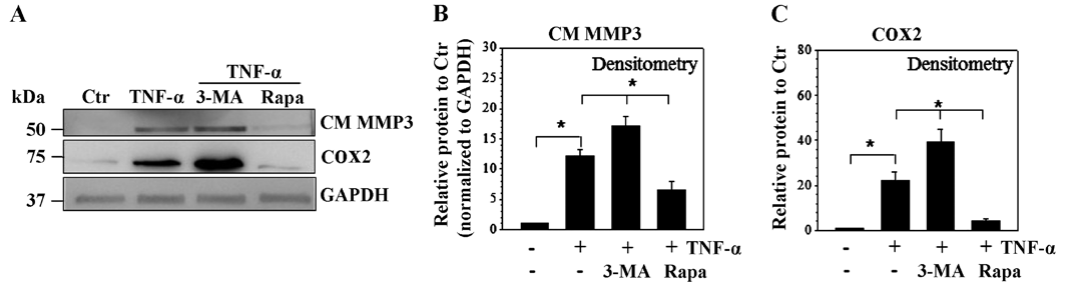
Autophagy regulates the catabolic effects induced by tumor necrosis factor-α (TNF-α) in nucleus pulposus (NP) cells at the protein level. (A) Western blot analysis showed cell protein COX2, condition medium protein and matrix metalloproteinase 3 (MMP3) expression in NP cells treated with autophagy inhibitor and activator. Densitometry showed protein expression of medium (B) MMP3 protein and (C) COX2 induced by TNF-α was significantly promoted by 3-methyladenine (3-MA) and markedly repressed by rapamycin (Rapa) in NP cells. Data represent mean + standard error of three independent experiments. ^*^P<0.05. CM, condition medium; Ctr, control.

To confirm the effect of autophagy on the catabolic effect induced by cytokines, western blot analysis was used to demonstrate the protein expression of the catabolic factors. The results showed that following TNF-α treatment the expression of medium protein, MMP3, and cell protein, COX2, increased to ~12- and 22-fold, respectively. Similar to the mRNA results, additional rapamycin repressed the expression to ~6- and 4-fold, respectively. However, the autophagy inhibitor, 3-MA, significantly upregulated the protein expression to ~18- and 39-fold, respectively (Fig. 3A–C).

### Autophagy is not regulated by TNF-α and IL-1β in rat NP cells

**Figure 4:**
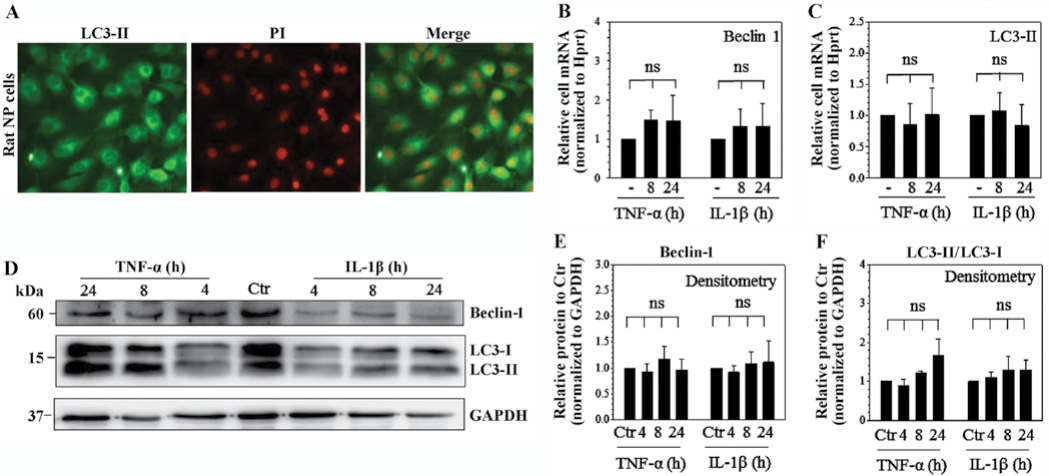
Autophagy of nucleus pulposus (NP) cells is refractory to tumor necrosis factor-α (TNF-α) and interleukin-1β (IL-1β) stimulation. (A) Rat nucleus pulposus (NP) cells cultured with serum-free medium showed robust expression of LC3-II in cytoplasm (magnification, ×20). Rat NP cells were treated with TNF-α or IL-1β, and (B) Beclin-1 and (C) LC3-II mRNA showed no change in response to cytokine treatment. (D) Western blot analysis and corresponding (E and F) densitometric analyses of NP cells treated with TNF-α and IL-1β. No significant change was observed in (E) Beclin-1 protein and (F) LC3 protein turnover. Data represent mean + standard error of three independent experiments. ^*^P<0.05. PI, propidium iodide; ns, not significant; Ctr, control.

There are numerous methods to identify the presence of autophagy in the cultured experimental cells. Among them, electron microscopy, LC3 turnover (LC3-I to LC3-II) and LC3-II expression are frequently used. LC3B immunofluorescence, acridine orange, monodansylcadav-erine staining and other methods are auxiliary methods to identify the presence of autophagy (29–35). To determine the effect of TNF-α and IL-1β on the autophagy of NP cells, immunofluorescence, RT-qPCR and western blot analyses were performed. Immunofluorescence microscopy showed the LC3-II expressed in rat NP cells (Fig. 4A) and no difference was observed following treatment with TNF-α and IL-1β. RT-qPCR and western blot analysis showed 8–24 h TNF-α and IL-1β treatment had no significant effect on the mRNA expression of Beclin-1, a marker of early phagophore formation or autophagy initiation, and LC3-II, the categorical autophagy-specific marker and indicator of autophagosome maturation (Fig. 4B and C). In addition, the Beclin-1 and LC3-II protein turnover showed no significant change after 4–24 h of TNF-α and IL-1β stimulation (Fig. 4D–F).

### Autophagy of rat NP cells is induced by NF-κB and JNK inhibition in inflammatory conditions

**Figure 5:**
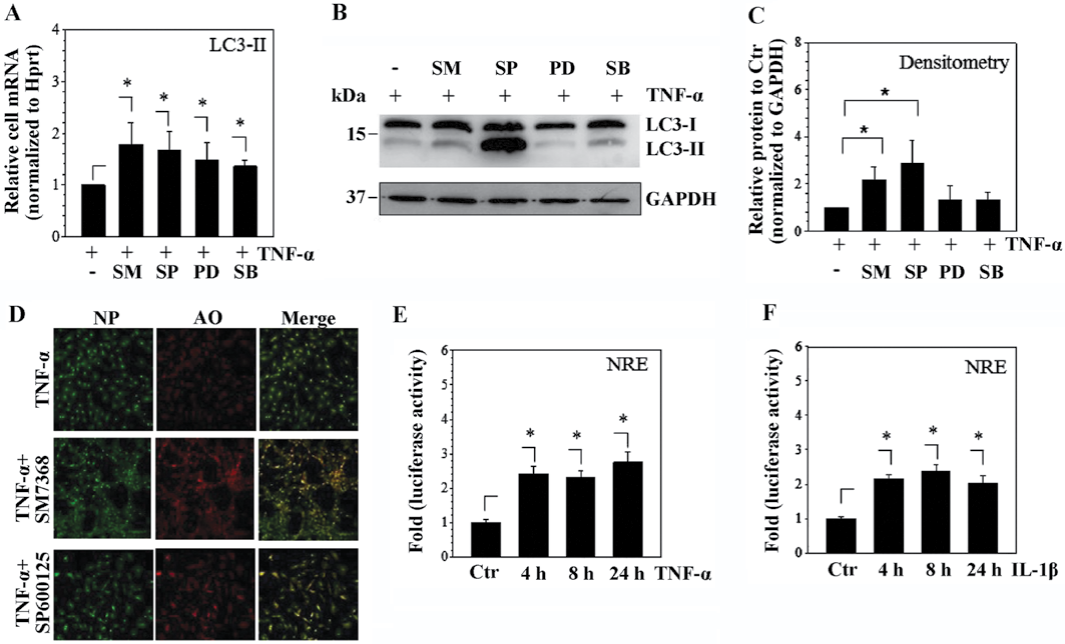
Autophagy of nucleus pulposus (NP) cells is activated by the inhibition of the nuclear factor κB (NF-κB) and c-Jun N-terminal kinase (JNK) signaling pathway in inflammatory conditions. (A) Rat NP cells were treated with tumor necrosis factor-α (TNF-α) and inhibitors; LC3-II mRNA significantly increased in response to SM7368, PD98059, SP600125 or SB203580 in inflammatory conditions. (B) Western blot analysis and (C) corresponding densitometric analyses of NP cells treated with TNF-α and inhibitors showed that only SM7368 and SP600125 increased the protein LC3-II expression. (D) Acridine orange staining (AO) showed more autophagic vacuoles in the cells treated with SM7368 compared with the control. NP cells were transfected with NF-κB promoter construct and treated with cytokines. (E) TNF-α and (F) interleukin-1β (IL-1β) increased the activity of NF-κB promoter construct. Data represent mean + standard error of three independent experiments. SM, SM7368; PD, PD98059; SP, SP600125; SB, SB203580; Ctr, control; NRE, NF-κB promoter construct. ^*^P<0.05.

To investigate the molecular mechanism of NP cell autophagy in inflammatory conditions, TNF-α was used to mimic the inflammatory condition and the NP cells were treated with NF-κB or MAPK inhibitors prior to TNF-α. SM7368, PD98059, SP600125 and SB203580 significantly increased the Beclin-1 and LC3-II mRNA expression in inflammatory conditions (Fig. 5A). However, only SM7368 and SP600125 treatment increased the LC3 protein turnover (LC3-II/LC3-I) (Fig. 5B and C). To further verify the effect of the NF-κB and JNK inhibitor, acridine orange staining was used to detect the autophagosome formation following SM7368 or SP600125 stimulation. The data showed that SM7368 or SP600125 treatment increased the autophagosome formation (Fig. 5D). To verify the efficacy of cytokines TNF-α and IL-1β on rat NP cells, the activity of the NF-κB promoter construct was measured following cytokine stimulation. As expected, the results showed that TNF-α or IL-1β significantly increased the activity of the NF-κB promoter construct (Fig. 5E and F).

### Autophagy of NP cells is induced by IKKβ knockdown

**Figure 6:**
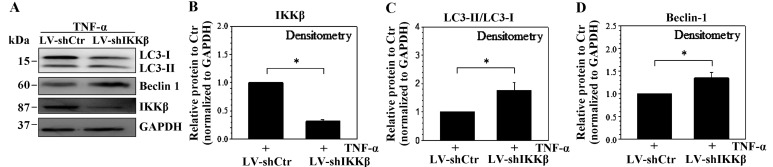
Stable silencing of IKKβ increases the autophagy-related protein expression in human nucleus pulposus (NP) cells. (A) Western blot analysis showed that the Beclin-1, LC3-II and IKKβ protein expression in NP cells was transduced with LV-shCtr or LV-shIKKβ in the tumor necrosis factor-α (TNF-α) condition. (B) Densitometric analyses of NP cells transduced with LV-shCtr or LV-shIKKβ; IKKβ expression levels were suppressed by LV-shIKKβ in the TNF-α condition. Compared to cells transduced with LV-shCtr, NP cells transduced with LV-shIKKβ demonstrated a higher expression of (C) LC3 protein turnover and an increase of (D) Beclin-1 protein in TNF-α condition. Data represent mean + standard error of three independent experiments. ^*^P<0.05. Ctr, control.

To further confirm the involvement of the NF-κB signaling pathway in controlling NP cells autophagy, an IKKβ loss-of-function study in TNF-α conditions using shRNA transduction was performed. As expected, compared to the transduction with control shRNA, densitometric analysis showed transduction with shIKKβ led to ~80% decrease of IKKβ protein in TNF-α conditions (Fig. 6A and B). Accordingly, transduction with shIKKβ in human NP cells resulted in a significant increase of the LC3 protein turnover and Beclin-1 protein in the inflammatory conditions (Fig. 6C and D).

## Discussion

The experiments described in the present investigation demonstrated that autophagy activation suppressed, while autophagy inhibition promoted, the catabolic effects induced by TNF-α and IL-1β. In addition, although autophagy-related mRNA and protein expression in NP cells was refractory to TNF-α and IL-1β, NF-κB and JNK inhibition increased the autophagy expression in inflammatory conditions.

Proteoglycan degradation contributes to the pathogenesis of IVD degeneration. TNF-α and IL-1β, significant inflammatory cytokines in IVDs, increased the proteoglycan degradation through the regulation of catabolic factors, such as MMP3, MMP9, ADAMTS4, ADAMTS5 and COX2 (7,36). In chondrocyte cells, autophagy suppressed the expression of catabolic factors induced by TNF-α and IL-1β (16,17). The present data also showed that TNF-α and IL-1β increased the mRNA expression of catabolic factors, *MMP3*, *MMP9*, *ADAMTS4* and *COX2*. Additionally, autophagy repressed the effect of TNF-α and IL-1β on *MMP3*, *MMP9*, *ADAMTS4* and *COX2* mRNA expression. Autophagy also suppressed *MMP2* mRNA expression in inflammatory conditions. Furthermore, the present results demonstrated autophagy inhibition could promote, while autophagy activation could repress, the catabolic effect of TNF-α on MMP3 and COX2 protein expression. Therefore, autophagy decreased the effect of cytokines, TNF-α and IL-1β, and thus, autophagy should be a protective factor in the process of IVD degeneration.

Of note, although TNF-α and IL-1β increased the proteoglycan degradation (7,36), autophagy was induced by TNF-α and IL-1β in chondrocytes, AF cells, fibrosarcoma L929 cells and breast cancer cells (14,16,18,30). The present data showed that, as opposed to in chondrocyte or cancer cells, the mRNA and protein expression of autophagy-related genes, LC3 and Beclin-1, showed no change in response to TNF-α and IL-1β. Therefore, cytokines appeared to be less critical for autophagy in NP cells compared with the other types of cells.

Inflammatory conditions were present in degenerative discs (37). Ye *et al* (18) showed that NF-κB and p38 inhibitor enhanced the TNF-α-induced autophagy in inflammatory conditions. Djavaheri-Mergny *et al* (38) also demonstrated that NF-κB activation represses TNF-α-induced autophagy. However, Copetti *et al* (39) showed that NF-κB can induce autophagy by transactivating Beclin-1. The autophagy regulation by MAPK is similar to NF-κB (19,40,41). The present data showed that in the inflammatory conditions, NF-κB, JNK, ERK and P38 MAPK inhibitor upregulated LC3-II mRNA expression; however, only the NF-κB and JNK inhibitor promoted the LC3 protein turnover. Furthermore, IKKβ loss of function experiments showed knockdown of *IKKβ* led to the increase of LC3 protein turnover even though TNF-α was present. Taken together, the NF-κB and JNK signaling pathway inhibition sustained the autophagy of NP cells in the presence of TNF-α.

In conclusion, inhibition of autophagy could diminish, and activation of autophagy could enhance, the catabolic effect of cytokines. Although autophagy is refractory to cytokines in NP cells, the NF-κB and JNK signaling pathway inhibition sustained the autophagy of NP cells in inflammatory conditions.

## References

[1] Luoma K, Riihimäki H, Luukkonen R, Raininko R, Viikari-Juntura E, Lamminen A (2000). Low back pain in relation to lumbar disc degeneration. Spine.

[2] Pockert AJ, Richardson SM, Le Maitre CL, Lyon M, Deakin JA, Buttle DJ, Freemont AJ, Hoyland JA (2009). Modified expression of the ADAMTS enzymes and tissue inhibitor of metalloproteinases 3 during human intervertebral disc degeneration. Arthritis Rheum.

[3] Séguin CA, Pilliar RM, Roughley PJ, Kandel RA (2005). Tumor necrosis factor-alpha modulates matrix production and catabolism in nucleus pulposus tissue. Spine.

[4] Studer RK, Gilbertson LG, Georgescu H, Sowa G, Vo N, Kang JD (2008). p38 MAPK inhibition modulates rabbit nucleus pulposus cell response to IL-1. J Orthop Res.

[5] Cui LY, Liu SL, Ding Y, Huang DS, Ma RF, Huang WG, Hu BS, Pan QH (2007). IL-1beta sensitizes rat intervertebral disc cells to Fas ligand mediated apoptosis in vitro. Acta Pharmacol Sin.

[6] Genevay S, Finckh A, Mezin F, Tessitore E, Guerne PA (2009). Influence of cytokine inhibitors on concentration and activity of MMP-1 and MMP-3 in disc herniation. Arthritis Res Ther.

[7] Tian Y, Yuan W, Fujita N, Wang J, Wang H, Shapiro IM, Risbud MV (2013). Inflammatory cytokines associated with degenerative disc disease control aggrecanase-1 (ADAMTS-4) expression in nucleus pulposus cells through MAPK and NF-κB. Am J Pathol.

[8] Ogier-Denis E, Codogno P (2003). Autophagy: A barrier or an adaptive response to cancer. Biochim Biophys Acta.

[9] Klionsky DJ, Emr SD (2000). Autophagy as a regulated pathway of cellular degradation. Science.

[10] Scheper W, Nijholt DA, Hoozemans JJ (2011). The unfolded protein response and proteostasis in Alzheimer disease: Preferential activation of autophagy by endoplasmic reticulum stress. Autophagy.

[11] Caramés B, Hasegawa A, Taniguchi N, Miyaki S, Blanco FJ, Lotz M (2012). Autophagy activation by rapamycin reduces severity of experimental osteoarthritis. Ann Rheum Dis.

[12] Ye W, Xu K, Huang D, Liang A, Peng Y, Zhu W, Li C (2011). Age-related increases of macroautophagy and chaperone-mediated autophagy in rat nucleus pulposus. Connect Tissue Res.

[13] Ye W, Zhu W, Xu K, Liang A, Peng Y, Huang D, Li C (2013). Increased macroautophagy in the pathological process of inter-vertebral disc degeneration in rats. Connect Tissue Res.

[14] Shen C, Yan J, Jiang LS, Dai LY (2011). Autophagy in rat annulus fibrosus cells: Evidence and possible implications. Arthritis Res Ther.

[15] Ma KG, Shao ZW, Yang SH, Wang J, Wang BC, Xiong LM, Wu Q, Chen SF (2013). Autophagy is activated in compression-induced cell degeneration and is mediated by reactive oxygen species in nucleus pulposus cells exposed to compression. Osteoarthritis Cartilage.

[16] Lin NY, Beyer C, Giessl A, Kireva T, Scholtysek C, Uderhardt S, Munoz LE, Dees C, Distler A, Wirtz S (2013). Autophagy regulates TNFα-mediated joint destruction in experimental arthritis. Ann Rheum Dis.

[17] Sasaki H, Takayama K, Matsushita T, Ishida K, Kubo S, Matsumoto T, Fujita N, Oka S, Kurosaka M, Kuroda R (2012). Autophagy modulates osteoarthritis-related gene expression in human chondrocytes. Arthritis Rheum.

[18] Ye YC, Yu L, Wang HJ, Tashiro S, Onodera S, Ikejima T (2011). TNFα-induced necroptosis and autophagy via supression of the p38-NF-κB survival pathway in L929 cells. J Pharmacol Sci.

[19] Xu P, Das M, Reilly J, Davis RJ (2011). JNK regulates FoxO-dependent autophagy in neurons. Genes Dev.

[20] Sommermann TG, Mack HI, Cahir-McFarland E (2012). Autophagy prolongs survival after NFκB inhibition in B-cell lymphomas. Autophagy.

[21] Kim JE, You DJ, Lee C, Ahn C, Seong JY, Hwang JI (2010). Suppression of NF-kappaB signaling by KEAP1 regulation of IKKbeta activity through autophagic degradation and inhibition of phosphorylation. Cell Signal.

[22] Colleran A, Ryan A, O'Gorman A, Mureau C, Liptrot C, Dockery P, Fearnhead H, Egan LJ (2011). Autophagosomal IkappaB alpha degradation plays a role in the long term control of tumor necrosis factor-alpha-induced nuclear factor-kappaB (NF-kappaB) activity. J Biol Chem.

[23] Li DD, Wang LL, Deng R, Tang J, Shen Y, Guo JF, Wang Y, Xia LP, Feng GK, Liu QQ (2009). The pivotal role of c-Jun NH2-terminal kinase-mediated Beclin 1 expression during anticancer agents-induced autophagy in cancer cells. Oncogene.

[24] Comes F, Matrone A, Lastella P, Nico B, Susca FC, Bagnulo R, Ingravallo G, Modica S, Lo Sasso G, Moschetta A (2007). A novel cell type-specific role of p38alpha in the control of autophagy and cell death in colorectal cancer cells. Cell Death Differ.

[25] Tang G, Yue Z, Talloczy Z, Hagemann T, Cho W, Messing A, Sulzer DL, Goldman JE (2008). Autophagy induced by Alexander disease-mutant GFAP accumulation is regulated by p38/MAPK and mTOR signaling pathways. Hum Mol Genet.

[26] Wang SC, Lin XL, Li J, Zhang TT, Wang HY, Shi JW, Yang S, Zhao WT, Xie RY, Wei F (2014). MicroRNA-122 triggers mesenchymal-epithelial transition and suppresses hepatocellular carcinoma cell motility and invasion by targeting RhoA. PLoS One.

[27] Pfirrmann CW, Metzdorf A, Zanetti M, Hodler J, Boos N (2001). Magnetic resonance classification of lumbar intervertebral disc degeneration. Spine.

[28] Ye W, Zhou J, Markova DZ, Tian Y, Li J, Anderson DG, Shapiro IM (2015). novel regulation by AP-1, Sp1, and Sp3. Am J Pathol.

[29] Paglin S, Hollister T, Delohery T, Hackett N, McMahill M, Sphicas E, Domingo D, Yahalom J (2001). A novel response of cancer cells to radiation involves autophagy and formation of acidic vesicles. Cancer Res.

[30] Wang J, Kim TH, Ahn MY, Lee J, Jung JH, Choi WS, Lee BM, Yoon KS, Yoon S, Kim HS (2012). Sirtinol, a class III HDAC inhibitor, induces apoptotic and autophagic cell death in MCF-7 human breast cancer cells. Int J Oncol.

[31] Mizushima N (2004). Methods for monitoring autophagy. Int J Biochem Cell Biol.

[32] Caramés B, Kiosses WB, Akasaki Y, Brinson DC, Eap W, Koziol J, Lotz MK (2013). Glucosamine activates autophagy in vitro and in vivo. Arthritis Rheum.

[33] Chen JW, Ni BB, Li B, Yang YH, Jiang SD, Jiang LS (2014). The responses of autophagy and apoptosis to oxidative stress in nucleus pulposus cells: Implications for disc degeneration. Cell Physiol Biochem.

[34] Cao Y, Yang W, Tyler MA, Gao X, Duan C, Kim SO, Aronson JF, Popov V, Takahashi H, Saito H (2013). Noggin attenuates cerulein-induced acute pancreatitis and impaired autophagy. Pancreas.

[35] Mizushima N, Yoshimori T, Levine B (2010). Methods in mammalian autophagy research. Cell.

[36] Millward-Sadler SJ, Costello PW, Freemont AJ, Hoyland JA (2009). Regulation of catabolic gene expression in normal and degenerate human intervertebral disc cells: Implications for the pathogenesis of intervertebral disc degeneration. Arthritis Res Ther.

[37] Vo NV, Hartman RA, Yurube T, Jacobs LJ, Sowa GA, Kang JD (2013). Expression and regulation of metalloproteinases and their inhibitors in intervertebral disc aging and degeneration. Spine J.

[38] Djavaheri-Mergny M, Amelotti M, Mathieu J, Besançon F, Bauvy C, Souquère S, Pierron G, Codogno P (2006). NF-kappaB activation represses tumor necrosis factor-alpha-induced autophagy. J Biol Chem.

[39] Copetti T, Bertoli C, Dalla E, Demarchi F, Schneider C (2009). p65/RelA modulates BECN1 transcription and autophagy. Mol Cell Biol.

[40] Sivaprasad U, Basu A (2008). Inhibition of ERK attenuates autophagy and potentiates tumour necrosis factor-alpha-induced cell death in MCF-7 cells. J Cell Mol Med.

[41] Jia G, Cheng G, Gangahar DM, Agrawal DK (2006). Insulin-like growth factor-1 and TNF-alpha regulate autophagy through c-jun N-terminal kinase and Akt pathways in human atherosclerotic vascular smooth cells. Immunol Cell Biol.

